# Adaptive memory and evolution of the human naturalistic mind: Insights from the use of medicinal plants

**DOI:** 10.1371/journal.pone.0214300

**Published:** 2019-03-26

**Authors:** Risoneide Henriques da Silva, Washington Soares Ferreira Júnior, Patrícia Muniz de Medeiros, Ulysses Paulino Albuquerque

**Affiliations:** 1 Programa de Pós-Graduação em Botânica, Universidade Federal Rural de Pernambuco, Recife, Pernambuco, Brazil; 2 Laboratório de Ecologia e Evolução de Sistemas Socioecológicos (LEA), Centro de Biociências, Departamento de Botânica, Universidade Federal de Pernambuco, Recife, Pernambuco, Brazil; 3 Universidade de Pernambuco, Vila Eduardo, Petrolina, Pernambuco, Brazil; 4 Grupo de Etnobiologia e Ecologia Humana, Centro de Ciências Agrárias, Universidade Federal de Alagoas, Rio Largo, Alagoas, Brazil; University of St Andrews, UNITED KINGDOM

## Abstract

Throughout evolutionary history, humans have been exposed to a wide variety of diseases, some of which have serious and even lethal consequences. Memorizing medicinal plants for the treatment of serious diseases likely maximized the chances of survival and reproduction and was instrumental in the evolutionary success of our species. In the present study, we used the idea of adaptive memory to understand whether human memory evolved to recall information about medicinal plants for the treatment of serious diseases. We considered plant-disease pairs of words as units of information available in a medical system based on the use of medicinal plants. The pairs included in the categories of chronic infectious diseases and transmissible infectious diseases were considered to be of higher adaptive value, whereas those included in the category of common conditions were considered to be of lower adaptive value. Pairs grouped into the category of emerging and reemerging diseases were employed to investigate conformity bias; pairs belonging to the category esthetic uses were considered to be of little adaptive relevance and utilized as an experimental control. Our results revealed that plant-disease pairs associated with the category of common conditions, considered by us to be of lower severity and less adaptive relevance for humans, were better remembered and retained in the participants' memory. We believe that prior experience with common conditions and the frequency of these conditions in the population may have intensified the ability to remember the plant-disease pairs associated with this group of diseases.

## Introduction

In response to the adversities faced by our ancestors, humans have better developed mechanisms to learn and retain information related to survival and reproduction than information that is not relevant to our fitness [1, 2, 3, 4, 5, 6, 7, 8, 9, 10].

From this perspective, the term adaptive memory was proposed by Nairne, Thompson and Pandeirada [1] to describe the differential performance of the human mind in a survival situation. For example, in one of their experiments, these authors presented students with three distinct scenarios: survival, moving and pleasantness. In the first scenario—survival—participants had to imagine themselves in a foreign land without basic subsistence supplies such as water and food and with the need to avoid predators. In this condition, participants were shown a list of words and asked to assess which of them would be relevant or irrelevant in dealing with the survival condition. In the second condition—moving, which was not related to the survival condition—participants had to imagine that they were moving to a new home in a foreign land, and they were instructed to assess the relevance of each presented word for locating and buying a house and carrying their belongings. In the third condition—pleasantness, which was used by the authors as a deep memory processing control—participants were shown a list of words and instructed to rate the pleasantness of each word. Some words could be pleasant, and others could not. The authors used Likert scales for all conditions. The results of the study showed that after the evaluation and/or classification of the words, followed by a revelation recall test, there was a greater retention of words relevant for the survival condition than of words relevant for the other conditions.

Aslam and Bauml [6] and Yang, Lau and Truong [8] replicated the results obtained by Nairne, Thompson and Pandeirada [1], and experiments using other stimuli, such as images and graphs, instead of words obtained similar results [11–12–13]. The same memory mechanisms that favor adaptive information have also been observed in people of different age groups and environmental contexts [14–17]. Studies have also revealed that people better remember animate stimuli (e.g., baby and lion) than inanimate stimuli (e.g., table and mountain) [18–19–20]. These findings reinforce the adaptive role of memory, as better recall of animated stimuli may have enabled our ancestors to avoid predators and potential enemies, as well as to identify partners and locate food, increasing the chances of survival and reproduction.

Fernandes et al. [13] suggested that human memory may be equipped to recall adaptive information relevant to health care. In one of their experiments, these authors showed the participants pictures of everyday objects along with a description of the health condition of the person who touched the object. The description presented was associated with a sick or healthy person. Participants were asked to assess whether one of the types of people touched the object. Subsequently, the authors performed an unanticipated recall test in which participants had to recall the names of the objects presented. As predicted by Fernandes et al. [13], participants better remembered the names of objects that were described as having been touched by sick people—a source of potential contamination—than the objects that were described as having been touched by healthy people. Bonin et al. [21] replicated the findings of Fernandes et al. [13]. In their first experiment, these authors presented three scenarios to the participants. The first two scenarios related to the context of contamination, avoidance of a potentially infectious disease, where the first scenario represented an ancestral environmental context and the second scenario represented a modern environmental context. The third scenario represented a control situation, in which participants imagined that they were organizing a trip for a group of people as their tour guide. After assessing which words would be relevant or irrelevant for dealing with each situation, participants faced a distraction task, followed by a surprise recall test in which they were instructed to write the words that they remembered. The authors observed no difference in recall regarding words involving contamination conditions both in ancestral environments and in modern environments. However, the two scenarios did differ significantly from the control condition in terms of recall. This evidence suggests that human memory may be equipped to recall adaptive information to avoid and prevent diseases.

Nonetheless, the idea of a human memory apt to remember any type of adaptive information well must be relativized. Sandry et al. [22] showed that people do not equally remember adaptive information. These authors studied the memorization of words in different adaptive scenarios—survival, fear and phobia, partner selection, avoidance of incest, detection of cheaters, jealousy, infidelity and gaining or maintaining status—and observed that remembrance of words for the survival scenario surpassed that of other information also considered adaptive. The authors then explained that memory is hierarchical because it prioritizes certain adaptive information to the detriment of other information. In this case, if the human memory were a rigid system that always favored any adaptive information, then all these adaptive mechanisms would be expected to have similar remembrance levels.

In this context, the existence of a human memory capable of recalling adaptive information in a hierarchical way is an interesting approach to understanding the functioning of the naturalistic human mind when confronted with situations of risk, such as avoidance of diseases. “Naturalistic mind is an abstract social-ecological entity. The way that we view nature and seek to understand the natural world is a complex aspect of our cognitive structure. The naturalistic mind was shaped by a process of reciprocal influence, particularly during our biological and cultural evolution, because of the relevance of information on the natural world to our species” [23]. Therefore, a compelling question that may derive from the idea of adaptive memory involves the understanding of how memory biases can affect the interactions between people and their environment [24].

Throughout evolutionary history, humans have had to deal with a wide variety of diseases, which varied in the degree of seriousness [25–26]. In this case, from an evolutionary scenario, better recall of adaptive information—medical practices or procedures—to deal with serious diseases may have given humans greater chances of survival and reproductive success than did recall of adaptive information to deal with diseases of lesser severity. If this is the case, then memory would prioritize only certain information within a survival scenario (dealing with disease).

In this study, we evaluated the possible adaptation of the human memory to favor information that is relevant to the treatment of serious diseases. To this end, we used medical practices involving the use of medicinal plants as a medical research model and consider pairs of words—plant-disease—as information units available in a medical system based on the use of medicinal plants. Medical systems can be understood as cultural systems formed by a set of concepts and practices related to health and illness, including symptom interpretations, treatment strategies and therapeutic outcome evaluation [27–28].

To build our research model, we obtained the vernacular names of the five most popular Brazilian medicinal plants [29]; for each medicinal plant name, we listed three names of diseases, considering data on their severity provided by the World Health Organization (WHO). The pairs of words were grouped by the degree of seriousness of the disease. We considered the plant-disease pairs included in the categories of chronic and transmissible infectious diseases as information that confers a greater adaptive advantage to humans because they involve diseases responsible for the annual leading causes of death in the world. However, we grouped the pairs of words included in the category of common conditions as information linked to a lower severity and lower adaptive value for humans. In addition, we considered plant-disease pairs included in the category of emerging and reemerging diseases to investigate a possible conformity bias, and we included the esthetic uses-category pairs of low adaptive relevance as an experimental control condition.

To test the proposed model, we formulated the following hypotheses: H1—medicinal plants associated with the treatment of serious diseases are best remembered in short-term memory, and H2—medicinal plants intended for the treatment of serious diseases are better retained in long-term memory. Our predictions were that (i) plant-disease pairs included in the serious disease categories would be significantly better remembered immediately than the pairs included in the categories of less serious diseases, and (ii) plant-disease pairs included in the categories of serious diseases would be better retained in long-term memory to the detriment of the pairs included in the categories of minor diseases. Therefore, the present study aimed to provide important insights into how the human mind may have evolved to remember and retain in memory adaptive information of human medical systems based on the use of medicinal plants.

## Methods

### 2.1 Experiment

#### 2.1.1 Participants

The study was conducted with 200 undergraduate and graduate students from the Federal Rural University of Pernambuco, Northeast Brazil, including 107 women and 93 men aged between 18 and 51 years (average = 23 and 17 years, respectively; standard deviation (SD) = 5 and 7, respectively).

The students were recruited through an online form available on the Survey Monkey digital platform. The forms’ access links were made available on the official website of the Federal Rural University of Pernambuco and on social networks linked to the institution. All students who completed the recruitment form and agreed to participate in the survey were contacted via email, and the experiment was scheduled. The students who agreed to participate signed a free and informed consent form, according to instructions of Resolution no. 466/12 of the National Health Council. The study was approved by the Ethics Committee for Research with Human Beings at the University of Pernambuco (Decision no. 1.714.676).

#### 2.1.2 Selection of plants and diseases

The vernacular names of five plants—mororó, cumaru, angico, mint and boldo—were selected from a list of 100 popular medicinal plants of Brazil, available from Medeiros, Ladio and Albuquerque [29]. For each plant, three diseases were chosen without replacement based on data from the WHO. The diseases were grouped into five categories: chronic diseases (stroke, cancer and diabetes), infectious diseases (AIDS, tuberculosis and measles), emerging and reemerging diseases (dengue, Zika fever and cholera), common conditions (cold, colic and diarrhea), and experimental controls (cellulitis, stretch marks and warts) (Table 1).

**Table 1:** Classification of plant-disease pairs in the categories of serious diseases—chronic and infectious transmissible diseases, low severity—common conditions and emerging and reemerging diseases, investigation of a conformity bias, and an experimental control–esthetic uses–of little adaptive relevance.

Disease category	Plant-disease
**Chronic diseases**	Mororó-Stroke Cumaru-Cancer Angico-Diabetes
**Infectious diseases**	Mint-AIDS Cumaru-Tuberculosis Boldo-Measles
**Common conditions**	Mororó-Cold Boldo-Colic Mint-Diarrhea
**Emerging and reemerging diseases**	Cumaru-Dengue Angico-Zika Mororó-Cholera
**Control**	Boldo-Cellulitis Mint-Stretch marks Angico-Warts

In this study, plants and diseases were considered units of information available in a medical system that is based on the use of medicinal plants. The repetition of the names of the same medicinal plants in different categories of diseases (Table 1) had the purpose of controlling whether "plant-disease" or information on the medical plants or diseases in isolation was the information that would be prioritized in the participants' memory.

For each category, different adaptive values were assigned according to disease severity. For example, the plant-disease pairs included in the categories chronic and transmissible infectious diseases were considered to have greater adaptive value due to their high severity and the risk of death that they pose to humans. According to the WHO, communicable chronic and infectious diseases are the annual leading causes of death worldwide and account for 36 and 14.2 million deaths, respectively [30].

We considered the plant-disease pairs of less adaptive relevance to be those included in the common conditions category; these diseases are of lower severity and risk to humans than chronic and infectious transmissible diseases. According to data from the United Nations International Children’s Emergency Fund (UNICEF) and the WHO [31], common conditions such as diarrhea are among the leading causes of death among children under five years of age and account for approximately 1.5 million deaths annually. The plant-disease pairs were included in the category of emerging and reemerging diseases to identify a possible conformity bias, that is, whether people would reproduce the most common information within the population [32–33] and would therefore tend to remember such information. For example, the plant-disease pairs associated with diseases currently disseminated in a social context, such as dengue and Zika fever, may be more memorable since they were frequently commented on by the media when data collection occurred. The plant-disease pairs associated with an experimental control—cosmetic uses—were considered to be of little adaptive relevance. In this study, we considered esthetic uses as a counterpoint to medical conditions; that is, these conditions would not lead to changes in the health status of the individual and therefore would not be important information to retain.

#### 2.1.3 Stimuli

The stimuli were composed of 15 plant-disease pairs printed on 15 cards made of paper. On each card, the vernacular name of a medicinal plant and its therapeutic target, separated by a hyphen, were printed. The pairs together totaled 30 words for memorization.

#### 2.1.4 Procedure

**Fig 1:**
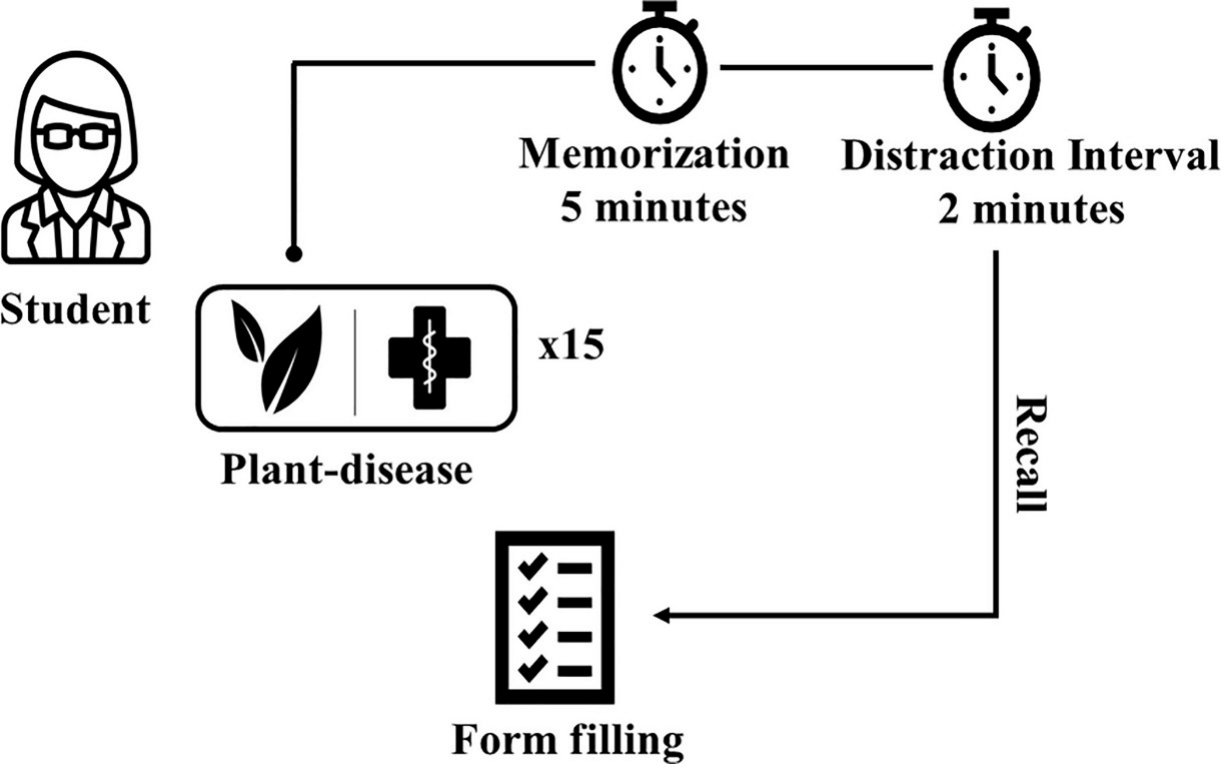
Procedure used for the memorization of plant-disease pairs of words by students of the Federal Rural University of Pernambuco, Brazil.

The experiment was divided into two phases. In phase I, the participant was individually tested in a 30-minute session and received 15 cards. Each card contained a plant-disease pair, and the cards were randomized before each session. The participants were instructed to read the information on the cards and memorize it for 5 minutes. Next, the cards were removed, and a distraction interval of 2 minutes was given, with the purpose of providing the time necessary to organize the information in the memory before the recall test. Thus, no mention was made of the recall test, and without warning, the memory was tested through free recall (Fig 1), which consisted of retrieving the plant-disease pairs that were randomly presented [34].

During free recall, the participant was instructed to complete a form with lines numbered one through fifteen, listing the plant-disease pairs in order of remembrance. This procedure was necessary for us to evaluate which pairs were first remembered by the participants. The form contained the following instructions: "*Be exact as possible*, *do not worry if you cannot remember all of the information*. *Fill in the blanks with the information you can remember*".

At the end of phase I, all participants were asked if they had previously known any of the plants that were used in the experiment. This procedure aimed to determine whether previous knowledge of the medicinal plants influenced the recall and retention of information. All aspects of the experiment were kept constant among participants. The memorization time for the plant-disease pairs was adapted from the protocol proposed by Nairne, Thompson and Pandeirada [1]. In this experiment, because the presentation of stimuli was not controlled by personal computers, 5 minutes was allowed for memorization. The duration of the experiment, the time of distraction, and the number of words used were identical to those in the original protocol.

Phase II of the experiment was conducted 30 days after phase I and began with an additional memory test, in this specific case to evaluate whether the information had been retained (long-term memory). The objective of this phase was to verify the information retained by the participants. At this stage, the two hundred students who participated in the first phase were recruited again. However, only 37 volunteers attended, and 24 of these volunteers were women. Each participant was individually tested and instructed to recall the information from the plant-disease pairs that were presented to them during the first phase of the experiment, now without the encouragement of the cards. The participants were also instructed to write down the recalled information on a form, again listing the pairs in order of remembrance. The form had the same structure as the one used in phase I.

#### 2.1.5 Data analysis

To test the prediction that the plant-disease pairs included in the categories of serious diseases would be recalled more frequently, the data were initially assessed using the Shapiro-Wilk test, which revealed that the data were not normally distributed. The nonparametric Kruskal-Wallis test was applied to verify differences in recalling the plant-disease pairs between the categories of major and minor diseases, followed by *Dunn's* post hoc test with a *Bonferroni* correction, which were used to identify differences in recall.

To perform the tests, the plant-disease pairs were ordered for each participant based on the individual recall lists. For example, the pairs for participant x were ordered as the first pair recalled, the second pair recalled, and so on. For each plant-disease pair, a score was assigned according to its recalled position. For example, a recalled pair in the first position received a score of 15, one in the second position received a score of 14, and so on. Pairs of words that were not remembered received a score of 0.

The plant-disease pairs were then organized by category of disease (group 1 = chronic diseases, group 2 = infectious diseases, group 3 = emergent and reemerging diseases, group 4 = common conditions, and group 5 = experimental control).

Simple linear regressions were performed with the purpose of verifying the existence of a causal relationship between the severity of the disease and the recall of the plant-disease pairs. The arithmetic averages of the scores assigned based on the recall position of each pair were extracted, and the pairs were then grouped into categories of major and minor disease severity.

Emerging and reemerging diseases were not considered in our regression analysis because these diseases were used to verify a possible conformity bias [32–33] and therefore did not fit into the major or minor severity categories defined in this study. After calculating the arithmetic means of the scores obtained for each of the remembered pairs, we observed that these data presented variance heterogeneity, and it was thus necessary to transform them with the Neperian logarithm. Next, the data were fit with a simple linear regression model to determine whether greater disease severity resulted in increased recall of the associated pairs.

To determine whether people would first recall the plant-disease pairs included in the serious disease categories, we used the cognitive salience index, which shows the psychological salience of a list of terms by combining the frequency and position at which these terms were remembered [35]. Based on the set of lists of plant-disease pairs remembered by each participant, the cognitive salience index was calculated by the ratio between the citation frequency of each pair (F) and the product of its average position in the lists (mP) and the total number of participants (N), according to the following formula: S = F/(N*mP), as proposed by Sutrop [35]. To examine whether the calculated salience values for each pairing were different than those expected by chance, a null distribution was created by using Monte Carlo simulations with 5,000 randomizations [36].

To determine whether prior knowledge of the medicinal plants influenced the recall, we removed all—plant-disease—pairs associated with medicinal plants that were declared as previously known by the participants from the data analysis. Subsequently, the Kruskal-Wallis test was applied to verify differences in the recall of the plant-disease pairs. Without the information related to previous knowledge, we also performed a simple linear regression with the arithmetic means of the scores obtained by each recalled pairing with the purpose of testing whether the severity of the disease was positively related to the recall of the associated pairs.

To evaluate the retention of information after 30 days, we performed the same tests. All of the abovementioned tests were performed using R [37], and the level of statistical significance was p <0.05. The recall error rate in our study was 71.4%, which was higher than that observed in other studies [13]. In contrast to other studies, our study evaluated the recall of the combined words rather than words in isolation, which could explain the higher error rate of our study.

## Results

### 3.1 Recall (phase I)

Our results showed differences in the recall of the plant-disease pairs of words (H = 95.612; p<0.001) among participants. With the application of *Dunn's* test a posteriori, we verified that the pairs of words included in the common conditions category were the ones that differed most in recall (Table 2). The mean recall for pairs of words in the category of common conditions (M = 4.74) was higher than that for pairs in the other disease categories (Table 3). A simple linear regression also showed that disease severity did not explain the recall of pairs of words (R^2^ = 0.08854; p>0.05; Akaike information criterion (AIC): 72.84417). We observed that the plant-disease pairs better remembered by the participants (Boldo-Colic, S = 0.26, p<0.001) were associated with the category of common conditions, which were considered in this study to be less severe diseases for humans. The descriptive statistics for this data set are shown in Table 3.

**Table 2:** Results of Dunn's multiple comparison test for differences in the recall of plant-disease pairs of words among disease categories (phase I).

	Chronic	Infectious diseases	Emerging and reemerging diseases	Common conditions
**Infectious diseases**	-5.49^(^***^)^	_	_	_
**Emerging and reemerging diseases**	-0.94^**(NS)**^	4.55^(^***^)^	_	_
**Common conditions**	-7.81^(^***^)^	-2.32^(NS)^	-6.87^(^***^)^	_
**Control**	-6.40^(^***^)^	-0.90^**(NS)**^	-5.46^(^***^)^	1.41^**(NS)**^

p<0.001***; p<0.01**; p<0.05*; NS = not significant.

**Table 3:** Means and SDs of recall of the plant-disease pairs of words (phase I).

Disease category	Mean general recall/ Disease category	Plant-disease	M	SD
**Chronic diseases**	2.17	Mororó-Stroke	0.9	3.20
		Cumaru-Cancer	3.78	5.81
		Angico-Diabetes	1.85	4.47
**Infectious diseases**	3.98	Mint-AIDS	7.95	6.66
		Cumaru-Tuberculosis	2.50	4.99
		Boldo-Measles	1.51	3.96
**Common conditions**	4.74*	Mororó-Cold	1.38	3.74
		Boldo-Colic	8.53*	6.39
		Mint-Diarrhea	4.31	5.94
**Emerging and Reemerging diseases**	2.46	Cumaru-Dengue	2	4.46
		Angico-Zika	4.61	6.10
		Mororó-Cholera	0.78	2.88
**Control**	4.27	Boldo-Cellulitis	4.30	5.91
		Mint-Stretch marks	5.94	6.41
		Angico-Warts	2.58	5.06

**M** = Mean; **SD** = standard deviation. p<0.05*

### 3.2 Retention (phase II)

The retention test of pairs of words was performed after a period of 30 days. The results showed differences in the retention of the plant-disease pairs (H = 13.239; p<0.01) among the participants, and we found through a Dunn's test that the pairs included in the category of common conditions differed the most in retention (Table 4). The mean retention observed for pairs of words in the category of common conditions (M = 1.53) was higher than that for pairs in the other disease categories (Table 5). In addition, a simple linear regression showed that disease severity did not explain the retention of pairs of words in phase II of the experiment (R^2^ = 0.1366; p> 0.05; AIC: 17.63363). We observed that the plant-disease pair that was best kept in the memory by the participants (Boldo-Colic, S = 0.41, p <0.001) was associated with the category common conditions, which contained diseases considered to be less severe. The descriptive statistics for this data set are shown in Table 5.

**Table 4:** Results of Dunn's multiple comparison test for differences in retention of the plant-disease pairs of words between disease categories (phase II).

	Chronic diseases	Infectious diseases	Emerging and reemerging diseases	Common conditions
**Infectious diseases**	-2.03^(**NS)**^	_	_	_
**Emerging and reemerging diseases**	-0.57^**(NS)**^	1.46^(NS)^	_	_
**Common conditions**	-3.15^(^**^)^	-1.12^(NS)^	-2.58^(^*^)^	_
**Control**	-2.24^(**NS)**^	-0.20^**(NS)**^	-1.67^(NS)^	0.91^**(NS)**^

p<0.001***; p<0.01**; p<0.05*; NS = not significant.

**Table 5:** Means and SDs of retention of the plant-disease pairs of words (phase II).

Disease category	Mean recall/ Disease category	Plant-disease	M	SD
**Chronic diseases**	0.11	Mororó-Stroke	-	-
		Cumaru-Cancer	-	-
		Angico-Diabetes	0.32	1.97
**Infectious diseases**	1.04	Mint-AIDS	2.03	5.20
		Cumaru-Tuberculosis	1.08	3.72
		Boldo-Measles	-	-
**Common conditions**	1.53*	Mororó-Cold	-	-
		Boldo-Colic	3.54*	6.34
		Mint-Diarrhea	0.70	2.98
**Emerging and Reemerging diseases**	0.36	Cumaru-Dengue	0.30	1.81
		Angico-Zika	0.78	3.33
		Mororó-Cholera	-	-
**Control**	1.06	Boldo-Cellulitis	1.65	4.26
		Mint-Stretch marks	1.54	4.49
		Angico-Warts	-	-

**M** = Mean; **SD** = standard deviation; (-) pairs not recalled. p<0.05*

Our results did not support the hypothesis that people better remember and retain information about medicinal plants associated with the treatment of serious diseases than information about other plants. We observed that plant-disease pairs included in the categories of lower-severity diseases were better remembered and retained in the memory of our participants than those in the other categories.

### 3.3 Previous experience with medicinal plants

To investigate the influence of previous experience with the medicinal plant during the recall of plant-disease pairs, we removed all pairs of words that were associated with previously known medicinal plants by the volunteers from the statistical analysis. When we removed these pairs, there were no differences in the recall of pairs of words (H = 4.8525; p>0.05) among participants, which was different from the result observed in phases I and II of this experiment. We also found that the severity of the disease did not explain the recall of pairs of words (R^2^: 0.02603; p>0.05; AIC: 38.66278), even after removing the pairs of words that were associated with the previously known medicinal plants. This fact revealed that the severity of the disease was not a determining factor for memory. Thus, it is likely that the observed differences in recall and retention of pairs of words during phases I and II of this study occurred due to the experience of the participants with the medicinal plants used in our study.

## Discussion

The results showed that within a hierarchy of major and minor illnesses, people tended to recall and retain the plant-disease pairs associated with the common conditions category of diseases, which in this study was considered to have reduced severity and adaptive relevance for humans. If memory was adapted to prioritize only information that promotes an increase in fitness, that is, increases the chances of survival and reproduction, then one would expect that plant-disease pairs associated with groups of severe diseases rather than pairs associated with groups of less severe diseases would be prioritized in the memory of individuals. However, this pattern was not what we observed.

Thus, it is possible that other mechanisms may act as a trigger for prioritization of information in memory. For example, some studies have indicated that previous experience with certain events [38–39–40] may shape the way that we perceive and prioritize certain information. Perceiving a risk as important and handling the risk may be more related to previous experience than to perceived severity [38–39–40].

Thus, inexperience with serious diseases may have led people to underestimate their risk and not prioritize in memory the plant-disease pairs associated with severe diseases. Some studies indicate that past experiences may be some of the primary motivators of risk perception and the likely concern with future events. For example, Gibbons and Groarke [41] observed that the previous experience of women with a history of breast cancer in the family significantly influenced their concern about cancer and, consequently, increased their risk perception of the disease.

Miceli, Sotgiu and Settanni [42] also found that high rates of risk perception in situations of vulnerability were more associated with experience of previous events than with the probability of a new occurrence. Thus, it is possible that the plant-disease pairs best remembered by the participants were those considered individually relevant since they may be related to a personal experience with the disease and could intensify the memory of this adaptive information.

Importantly, we observed that medicinal plants stated as previously known by participants appeared to play an important role and were prioritized in memory. This result may be evidence that the medicinal plants associated with the treatment of diseases with which people have previous experiences are better remembered than are those plants associated with the treatment of serious diseases. Notably, our results differed from the results of other studies since we compared information on plant-disease pairs at different levels of disease severity, unlike other studies that compared the recall of hazardous and nonhazardous information [43].

In addition, other studies have shown that the frequency at which certain events occur can also shape the way we prioritize certain information [38–39]. Thus, a lower frequency of severe disease occurrence in the population may have acted as an important factor for the non prioritization in the memory of plant-disease pairs associated with the categories of diseases of greater severity. For example, according to the Brazilian Ministry of Health [44], common conditions, such as diarrhea, are frequent among the Brazilian population and accounted for 4,380,256 cases in 2013 alone, whereas more serious diseases such as AIDS presented only 48,000 new cases in Brazil in 2016 [45].

Therefore, our assumption is consistent with the results reported in other studies. For example, Ruin, Gaillard and Lutoff [38] observed that some people perceive a rare event of great magnitude and serious consequence as posing a lower risk due to its infrequency. Sachs et al. [39] obtained similar results, which suggested that most of the patients in the study with diabetes focused their attention on less serious and more prevalent adverse events during treatment rather than on more serious and less prevalent adverse events.

Here, we show that previous experience with a disease and its frequency in the population can shape how we prioritize information in our memory for the treatment of diseases. Our findings suggest that human memory may be biased toward remembering medicinal plants associated with the treatment of frequent diseases in the population rather than those for the treatment of less frequent diseases.

Another important fact is that the plant-disease pairs associated with emerging and reemerging diseases were not prioritized, even though this information was highly disseminated by the media at the time when we began collecting data. This finding reinforces our argument that prior experience with illness may be one of the major enhancers of memory. For example, Alqahtani et al. [46] observed that emerging infectious diseases, such as Middle East respiratory syndrome and Ebola virus disease, which many people were susceptible to, were perceived as being of lower risk than recent mass catastrophes. This result demonstrates that previous experience with a given event may enhance the ability to memorize it.

Therefore, medicinal plants associated with frequent illnesses and previous experiences appear to play an important role in human memory and indicate that our memory system is flexible with respect to the recall of adaptive information. This result seems to be consistent with a hierarchy of human memory [22], in which some adaptive information, such as that associated with survival, is better remembered than other information. According to Sandry et al. [22], specific adaptive information appears to be intrinsically linked to our memory system. However, not all information that promotes the fitness of our species is easily recalled.

From an evolutionary perspective, our findings may also show certain characteristics that were selected in the past and shaped the human naturalistic mind. Within a model associated with survival (treatment of diseases), memory may also be hierarchized to prioritize adaptive information about highly frequent diseases and linked to previous experience. It may be that during the past, remembering a medicinal plant used to treat a common condition that consistently affected daily subsistence practices—for example, obtaining food, escaping from predators, protecting offspring or reproducing—could have been more relevant than remembering plants used to treat a serious disease of occasional occurrence.

If this relationship is not true, then the recall of information about plants associated with diseases that frequently affect people and that are heavily influenced by previous experiences may be further evidence that other mechanisms can shape the way we prioritize certain information in memory, independent of the role of the evolutionary past. For example, a recent study revealed the existence of a human preference for tropical forest landscapes, contrary to what was expected to be an innate human preference for savannah landscapes [47], an environment where the earliest humans probably evolved. This fact may indicate that other factors (e.g., cultural variables), rather than just the selective pressures of ancestral environments, are influencing with the functioning of the human mind.

Therefore, according to our findings, there may not be a requirement to recall information about medicinal plants used to treat diseases and that would make humans more likely to survive and reproduce, suggesting that there may be no ancestral priorities. Nonetheless, additional studies are needed to test such hypotheses. Deciphering which adaptive information receives prioritization in memory and the mechanisms that intensify such abilities can help us understand the evolution and function of our naturalistic minds. We suggest that future studies be guided by such inquiries and that new experimental designs be conceived.

## Conclusions

Humans can remember and retain information associated with the treatment of common conditions that we considered to be less severe in our study. It is possible that previous experiences with a disease and its frequency in the population shape how we prioritize and retain information. Therefore, human memory appears to be flexible in recalling adaptive information associated with medical practices involving plants, which is in contrast to our hypothesis that there is a priority for serious illnesses.

## Limitations of our study

Only a few participants were engaged in the second phase of the experiment, which may have occurred because we did not apply a stimulus (such as a cash contribution) that would motivate students to participate. However, this limitation did not interfere with our results. The differences in the number of participants in phases I and II were considered separately in our statistical analyses. The statistical analyses allowed us to predict possible differences in sample size during data analysis.

Unfortunately, we did not control for the effect of previous experience of an individual with a disease with regard to recall of disease-plant pairs of words. Although this limitation did not affect the interpretation of our findings that some pairs of words were better recalled than others, this variable should be controlled for in further studies. The use of esthetics as an experimental control may have led to bias because esthetic problems can affect health and psychological comfort. Thus, we suggest in future studies the use of an experimental control that allows greater neutrality.

## Supporting information

S1 Appendix Cards with pairs of words (plant-disease) and form used in the experiment. (DOCX)

S2 Appendix Dataset. (XLSX)
