# Supplementary material for: Adaptive memory and evolution of the human naturalistic mind: Insights from the use of medicinal plants
Source: PLoS One. 2019 Mar 26;14(3):e0214300. doi: 10.1371/journal.pone.0214300 (PMC6435313; doi:10.1371/journal.pone.0214300)
Supplement: S1 Appendix — (DOCX) [file pone.0214300.s001.docx]

**Adaptive memory and evolution of the human naturalistic mind: insights from the use of medicinal plants**

Risoneide Henriques da Silva, Washington Soares Ferreira Júnior, Patrícia Muniz de Medeiros, Ulysses Paulino Albuquerque

**Appendix A: Cards with the names of plant-disease pairs used in the experiment.**

**Chronic**

Angico-Diabetes

Cumaru-Cancer

Mororó-Stroke

**Infectious diseases**

Mint-AIDS

Cumaru-Tuberculosis

Boldo-Measles

**Common conditions**

Mint-Diarrhea

Boldo-Colic

Mororó-Cold

**Emerging and reemerging**

Mororó-Cholera

Cumaru-Dengue

Angico-Zika

**Control**

Mint-Stretch marks

Boldo-Cellulitis

Angico-Warts

**Appendix B: Form used by volunteers to list names of plant-disease pairs in order of recall.**

**Name: ________________________________________Code: ________**

"*Be exact as possible, do not worry if you cannot remember all of the information. Fill in the blanks with the information you can remember*".

1.____________________________________________2.____________________________________________3.____________________________________________4.____________________________________________5.____________________________________________6.____________________________________________7.____________________________________________8.____________________________________________9.____________________________________________10.___________________________________________11.___________________________________________12.___________________________________________13.___________________________________________14.___________________________________________15.__________________________________________________________________________________________________________________________________________________________________________________________________________________________________________________________________________________________________________________________________________________________________________________________________________________________________________________________________________________________________________________________________________________________________________________________________________________________________________________________________
